# Clinical characterization and management of persons with comorbid epilepsy and depression: an expert opinion paper

**DOI:** 10.3389/fphar.2025.1592650

**Published:** 2025-07-01

**Authors:** Gaia Sampogna, Roberto Brugnoli, Giuseppe Didato, Maurizio Elia, Edoardo Ferlazzo, Gianluca Serafini, Giampaolo Vatti, Umberto Volpe, Flavio Villani, Gabriele Sani

**Affiliations:** ^1^ Department of Psychiatry, University of Campania “L. Vanvitelli”, Naples, Italy; ^2^ Department of Psychiatry, Fondazione Policlinico Universitario Agostino Gemelli IRCCS, Rome, Italy; ^3^ Department of Neuroscience, Section of Psychiatry, Università Cattolica del Sacro Cuore, Rome, Italy; ^4^ Epilepsy Unit, Fondazione IRCCS Istituto Neurologico Carlo Besta, Milan, Italy; ^5^ Oasi Research Institute-IRCCS, Troina, Italy; ^6^ Department of Medical and Surgical Sciences, Magna Graecia University, Catanzaro, Italy; ^7^ Department of Neuroscience, Rehabilitation, Ophthalmology, Genetics, Maternal and Child Health (DINOGMI), Section of Psychiatry, University of Genoa, Genoa, Italy; ^8^ Unità Operativa Complessa Neurology and Clinical Neurophysiology, University Hospital of Siena, Siena, Italy; ^9^ Division of Clinical Neurophysiology and Epilepsy Center, Istituto di Ricovero e Cura a Carattere Scientifico Policlinico San Martino, Genoa, Italy; ^10^ Unit of Clinical Psychiatry, Department of Clinical Neurosciences/DIMSC, Polytechnic University of Marche, Ancona, Italy

**Keywords:** depression, epilepsy, comorbidity, burden, differential diagnosis

## Abstract

**Introduction:**

Medical conditions related to the nervous system affects 3.4 billion individuals worldwide and are collectively ranked as the leading cause of disability-adjusted life years (DALYs). Epilepsy is listed among the ten conditions with the highest age standardized DALYs, while depression is expected to become the first cause of disability for mental disorders by 2030. Persons with epilepsy have a higher risk of developing depression, and *vice versa*. Epilepsy and depression both can influence individual’s personal functioning, social activities and can increase the risk of sudden epileptic attacks. Depression is probably the most frequent psychiatric comorbidity reported in patients with epilepsy. Several studies have highlighted a bidirectional association between depression and epilepsy.

**Methods:**

The present mini-review is based on expert meetings held in the period May-September 2024. A panel of expert clinicians, working in the field of epilepsy and of clinical psychiatry have been invited to participate, due to their experience and expertise in the topic. Panel members, under the guidance of two senior experts, have identified the relevant topics to be analyzed, discussed and commented.

**Results:**

Included studies dealt with the historical perspective on epilepsy and depression; the complexity of diagnostic and clinical comorbidity between epilepsy and depression; the assessment tools for screening for depression in patients with epilepsy.

**Discussion:**

The clinical condition of patients suffering from comorbid depression and epilepsy represents a challenge for neurologists and psychiatrists. Therefore, the management of comorbid epilepsy and depression requires a detailed clinical characterization of each individual case in order to develop an integrated and personalized management plan.

## Introduction

Diseases affecting the nervous system represent the leading cause of disability-adjusted life years (DALYs), with 3.4 billion individuals suffering from these diseases. In the period 1990–2021, a 18.2% increase in the global DALY associated to such conditions have been recorded. Considering the top-ten conditions associated with the highest age-standardised DALYs, epilepsy ranked as first disease. Looking at mental disorders, major depression disorder (MDD) represents the third cause of burden of disease worldwide and it is expected to become the first cause of disability for mental disorders by 2030 (Beghi, 2020). Therefore, both neurological and psychiatric disorders are the leading causes of disability and death worldwide, explaining 14% of the global disease burden.

According to recent WHO estimates, more than 50 million people are affected worldwide from epilepsy, regardless of age, race, geographical region and socioeconomic status (GBD 2021 Nervous System Disorders Collaborators, 2024).

The personal, social and economic burden associated with epilepsy is significantly high, especially in terms of healthcare needs, premature death and work absenteeism and lost productivity. Moreover, patients suffering from epilepsy report high levels of stigma and discrimination, which hamper and delay the access to adequate treatments. People with epilepsy report a three times higher risk compared to general population of premature death. This mortality gap is particularly high in people suffering from epilepsy and living in low- and middle-income countries. A recent WHO report highlights that “up to 70% of people living with epilepsy could live seizure-free if properly diagnosed and treated” (Levira et al., 2017; Manole et al., 2023).

MDD represents the most common severe mental disorder, with more than 300 million of people affected worldwide. The 12-month and lifetime prevalence is of 13.4% and 26.1%, respectively, with the female population reporting a higher incidence compared to male population (14.7% vs. 7.2%, respectively) (Fischer et al., 2023; Salk et al., 2017). The incidence rates are even higher when considering special female population, such as pregnant women. In this target population, up to 10% of them who are pregnant and/or have just given birth experience depression (Sidhu et al., 2019; Fatori et al., 2020; Di Vincenzo et al., 2022; La Verde et al., 2024). Although effective treatments for MDD are available, more than 75% of people in low- and middle-income countries receive no treatment (Evans-Lacko et al., 2018; Tsigebrhan et al., 2023; Sakurai and Kanemoto, 2022). Barriers to effective care include a lack of investment in mental healthcare, lack of trained healthcare providers and social stigma associated with mental disorders (Volpe et al., 2014; Jain et al., 2023; Holt-Lunstad, 2024).

The scenario is even more complex when considering patients suffering from comorbid mental and nervous diseases, in particular suffering from epilepsy and depression. It has been repeatedly highlighted that people with epilepsy have a higher risk of developing depression, and viceversa (Kanner, 2014). Epilepsy and depression both can influence individual’s personal functioning, social activities and they can increase the risk of sudden epileptic attacks (Lee et al., 2018; Cronin et al., 2023).

In patients with epilepsy, the prevalence of depression ranges between 10.7% to 44%, with higher rates in patients with refractory epilepsy (up to 54%) (Qin et al., 2022). Depression represents the most common comorbid condition affecting people with epilepsy (Berk et al., 2023; Abe et al., 2020). The comorbid depressive disorder in people with epilepsy negatively impact on patients’ personal functioning and quality of life. Several studies have highlighted a bidirectional association between depression and epilepsy.

Based on these premises, the present mini-review has been conducted in order to clarify the complexity of comorbidity between epilepsy and depression as well as the main critical issues for the optimal clinical management of patients suffering from these conditions.

## Materials and methods

The present mini-review is based on expert meetings held in the period May-September 2024. A panel of expert clinicians, working in the field of epilepsy and of clinical psychiatry have been invited to participate, due to their experience and expertise in the topic.

Panel members, under the guidance of two senior experts, have identified the following topics to be analyzed, discussed and commented: a) historical perspective on epilepsy and depression; b) complexity of comorbidity between epilepsy and depression; c) complexity of clinical presentation of depression in patients with epilepsy; d) screening for depression in patients with epilepsy; e) management plan for persons with comorbid depression and epilepsy; f) unmet clinical needs in the management plan of patients with comorbid depression and epilepsy.

This paper provides an overview of the complex clinical presentation of depressive symptoms/depressive disorder in people suffering from epilepsy and aims to point out the most urgent clinical unmet needs that should be addressed in the next future.

The main areas of interest identified during the meetings are reported in the Results’ section. Updated research studies, as well systematic reviews and meta-analyses related to the clinical characterization and management of depression in people with epilepsy were selected. No date limits were applied. Only articles in English language were selected. The final reference list was generated based on novelty, importance, originality, quality, and relevance to the scope of this review.

## Results

### Historical perspectives on epilepsy and depression: the long journey of complex diseases toward a modern conceptualization

Epilepsy is considered one of the oldest clinical conditions recognized by medical community, with written records dating back to 4000 BCE (Magiorkinis et al., 2010). The term “epilepsy” derives from the Greek verb *epilambanein* (επιλαμβάνειν), meaning to be seized or take hold. Studies carried out by Hippocrates (in 400 BC) suggested a natural cause of epilepsy, rather than considering it a divine or sacred epiphenomenon. For the first time, Hippocrates suggested the relationship between epilepsy and depression, stating that “melancholics ordinarily become epileptics, and epileptics, melancholics: what determines the preference is the direction the malady takes; if it bears upon the body, epilepsy, if upon the intelligence, melancholy” (Lewis, 1934).

During the Middle Age, epilepsy and depression were considered as magical or mystical conditions, with people suffering from epilepsy considered as possessed. Such misconception still persists in several low- and middle-income countries, nurturing the stigma surrounding people with epilepsy and delaying the access to adequate care (Kaculini et al., 2021; Saha et al., 2017; Reed, 2024).

In the essay “Anatomy of Melancholy”, written by R. Burton in 1692, he identified several social and psychological factors as causes of depression, including poverty, fear, and loneliness. Subsequently, depressive disorders have been considered as a weakness in temperament, mainly on an heritable basis and–therefore–not amenable to change.

At the same time, the neurologist John Hughlings Jackson–considered as the founder of the modern epileptology–developed the first comprehensive explanation of seizure origin. He proposed the following definition: “Epilepsy is the name given for occasional, sudden, excessive, rapid and local discharges of grey matter”. Subsequently, William Richard Gowers, Jean Martin Charcot, Charles-Moïse Briquet and Bénédict Augustin Morel proposed to differentiate epileptic convulsions from nonepileptic (hysterical) convulsions (Faber, 1997).

In 1917, S. Freud published his conceptualization of depression and he described the concept of melancholia as “being a response to loss, either real (for example, a death) or symbolic (such as failure to achieve the desired goal)” (Freud, 1954).

In 1930s the studies by Hans Berger clarified the aetiopathogenesis of epilepsy, with the discovery of phenobarbital and the development of electroencephalogram (EEG). The localisation of epileptic discharges in the brain and their association with lesions was more important than anything else in determining the character of the seizures. Nevertheless, the clinical impression continued to exist that patients with epilepsy suffer from mental problems. In 1949, the discovery of the temporal lobe focus (and, therefore, of temporal lobe epilepsy), as well as the description of the limbic system, led to the idea of mental disorders being linked to epileptic disturbances in specific brain areas. People with epilepsy were considered mentally normal, but it was assumed that brain dysfunctions would lead to seizures as well as psychological manifestations (Bear, 1979).

Currently, it is believed that not only biological factors (e.g., aetiology, focus localisation), but also medication (e.g., number and types of medication such as phenobarbital, topiramate, brivaracetam, clobazam, levetiracetam, perampanel, vigabatrin and zonisamide) can negatively impact mood (Datta, 2023). Moreover, psychological and social factors (e.g., fear of seizures, perceived stigma) are important aspects in the development of mental disorders in patients suffering from epilepsy (Mendez et al., 1986).

According to the biopsychosocial model of mental disorders, it should be argued that the complex reorganization of brain circuits leading to the two conditions occurs in predisposed individuals and such a predisposition is due to a combination of genetic background and environmental factors (Maj et al., 2020; Maj, 2023; Fusar-Poli et al., 2023). This model could be useful for explaining why some patients develop only epilepsy or only depression or both depending on the individual combinations of predisposing factors and environmental contributors/stressors (Østergaard et al., 2023; Fiorillo and Giordano, 2022; Madigan et al., 2023).

### Focus on comorbidity between epilepsy and depression

Several factors should be considered for explaining the close inter-relation between epilepsy and depression. Epilepsy is a severe condition associated with high levels of stigma and discrimination. Furthermore, people suffering from epilepsy can experience limitation in their daily life (e.g., driving licence limitation/loss) as well as they have to cope with the unpredictable nature of epileptic seizures. All these factors can contribute to the poor level of self-esteem, social withdrawal, and demoralization.

People with epilepsy frequently suffer also from depression. This comorbid condition is often underdiagnosed and undertreated, due to several hampering factors including patients’ reluctance to complain about psychiatric symptoms as well as poor attention dedicated from neurologists to detect, recognize and manage mental disorders in comorbidity with epilepsy (Temple et al., 2023).

Depressive symptoms and poor levels of quality of life are reported in almost 30% of patients with epilepsy, with a higher incidence (up to 60%) in those patients suffering from drug-resistant epilepsy (Peltola et al., 2024; Robertson et al., 1987; Lambert and Robertson, 1999). Depression in epilepsy may lead to worse seizure outcome, significant functional and psychosocial disability, a higher rate of self-injurious behavior, and an increased risk of suicidal ideation and attempt (Nickels, 2021).

Although depression represents a common comorbid disorder in people with epilepsy, another reason for not treating it adequately is due to the misconception that it is a direct consequence of the disease itself or it is a medication-side effect.

Several socio-demographic and clinical factors have been identified for explaining the high risk of developing depression in people with epilepsy, including low-education level, unemployment, non-adherence with anti-seizure medications (ASMs), polypharmacological treatment, anxiety, stigma, discrimination and social isolation (Liu et al., 2024).

Furthermore, the presence of comorbid depression increases the risk of a refractory/difficult to treat form of epilepsy (Krishnan, 2020).

It has been found that up to 3 years prior to diagnosis of epilepsy, patients reported high levels of depressive and anxiety symptoms as well as of suicidal ideation (Bølling-Ladegaard et al., 2023). These findings highlight the complex pathophysiological mechanisms shared between depression and epilepsy (Galynker et al., 2024; Hesdorffer et al., 2012; Pompili et al., 2022).

As regards the neurobiological alterations in people with depression, it has been found that people report a significant bilateral reduction in hippocampal volumes, as well as a decreased cortical thickness in the frontal lobe, and decreased glial/neuronal cell density in the cingulate gyrus, rostral and caudal orbitofrontal cortex, and dorsal prefrontal cortex (Mula and Sander, 2019; Mula, 2019). The interesting finding is that also in patients suffering from chronic temporal lobe epilepsy, similar alterations can be highlighted. This should confirm–from a neurobiological viewpoint–the shared ethiopathogenesis of these clinical conditions (Mula and Sander, 2019). Moreover, studies based on animal models have clarified the role of serotonin dysfunction in patients with depression and with epilepsy. In mouse model characterized by a deletion of the 5-HTC2 receptor subunit, a lower seizure threshold for audiogenic seizures have been found, while in animal models for epilepsy and depression a reduced postsynaptic and increased presynaptic density of 5-HT1 receptors have been found (Brennan et al., 1997). Therefore, it should be argued that the alteration in serotonin neurotransmission can be responsible for the occurrence of both conditions (Maia et al., 2017; Guiard and Di Giovanni, 2015). This would also suggest that depression can represent a premorbid symptom in some epileptic syndromes.

Another neurobiological mechanism of depression, namely, the hyperactivation of the hypothalamic-pituitary-adrenal axis, is implicated also in epilepsy. The above-mentioned neurobiological pathways have been proposed for explaining the high comorbidity rates of epilepsy and depression, but no specific hypothesis can be considered conclusive (Mula and Sander, 2019).

### The complexity of presentation of depression in patients with epilepsy

The complexity of interrelationship between depression and epilepsy represents a challenge for clinicians in ordinary outpatient and inpatient setting. In a recent study by Shi et al. (2023), the prevalence of depression was 27.30% in patients with epilepsy. Gilliam et al. found that in neurological outpatient unit, screening for depressive symptoms/depressive disorder are performed only in 20% of cases (Gilliam et al., 2006; Gilliam, 2005). In particular, the clinical presentation of depressive symptoms in patients with epilepsy can be subtle and difficult to detect, since in most cases the presentation is atypical.

In fact, the clinical presentation of depression in epilepsy is multifaceted with many interacting neurobiological and psychosocial determinants. In particular, the presentation of depression can be influenced by clinical features of epilepsy, in terms of seizure frequency, type, foci, or lateralization of foci, neurochemical or iatrogenic mechanisms.

Specific type of epilepsy, such as the focal form and the bilateral tonic-clonic seizures have been identified as risk factors for developing a depressive disorder. Another clinical risk factor for depression in people with epilepsy is represented by the age: for each 1 year increment, the risk of developing depression increase by 3.8%. Nevertheless, up to 70% of patients with epilepsy did not receive any treatment for comorbidity (Shi et al., 2023). A significant gender-based difference in depression rates in patients with epilepsy have been found, with female patients 4.27 times more likely to suffer from depression than males. Risk factors for depression among female patients with epilepsy included type of occupational condition, years living with epilepsy, frequency/type of seizures, number of ASM used, and EEG findings. For male patients, the most relevant risk factors include age, ethnic group, and working condition (Guo et al., 2023). Furthermore, in female patients, suffering from epilepsy for less than 10 years, being treated with psychotropic drugs and reporting generalized seizure are additional risk factors for developing depression (Vacca et al., 2022).

#### Clinical presentation

In patients suffering primarily from epilepsy, the most common clinical presentation of depression includes fatigue, irritability, poor tolerance to frustration, anxiety, mood lability, and anhedonia. A study carried out in Italy found that the alteration of sleeping patterns, tiredness, and loss of energy were the most common symptoms of depression in people with epilepsy (Vacca et al., 2022).

**FIGURE 1:**
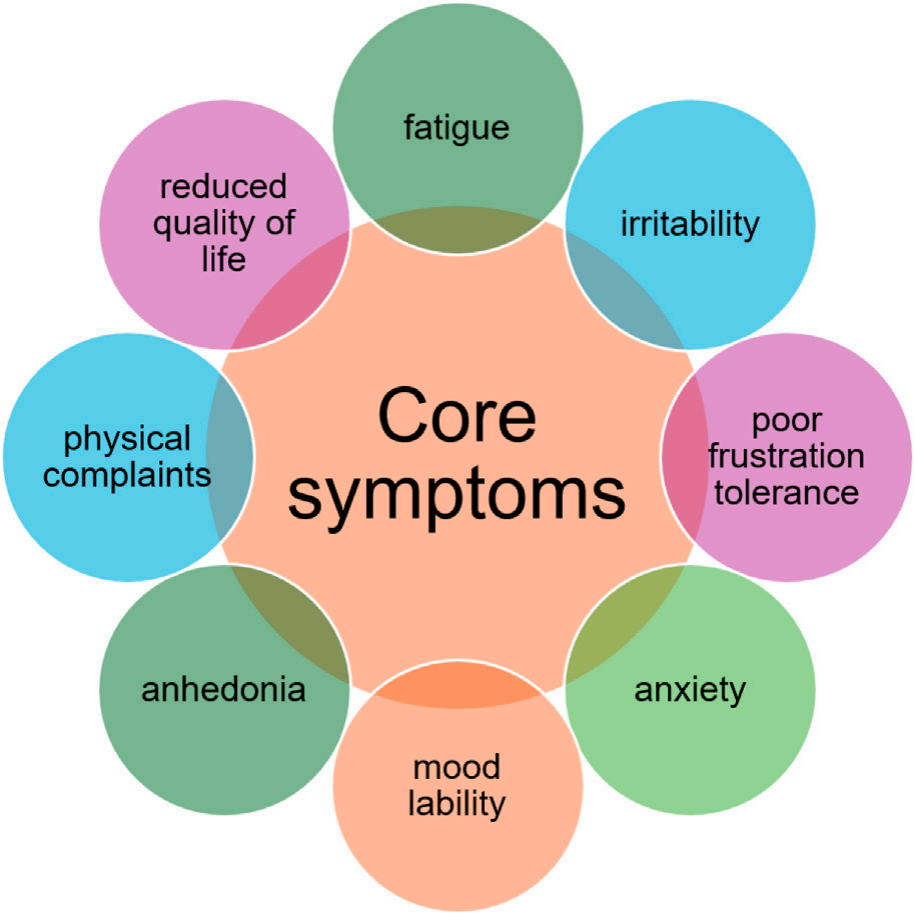
Clinical presentation of depression in patients with epilepsy.

Therefore, the “atypical” presentation of depressive disorders in people with epilepsy has been considered the most common type of depression (Figure 1).

In many specialized centres, the vast majority of cases where an underlying depressive disorder is detected, it is characterized by sadness, anxiety, thoughts of hopelessness/suicide, abulia, and insomnia. In other cases, depression is described as “atypical” due to the lack of training for healthcare professionals in detecting and treating such clinical conditions.

In the seminal works by Kraepelin and Bleuler, the heterogeneous presentation of depressive symptoms in patients with epilepsy was described. In particular, they highlighted that in those patients, depression was mainly characterized by irritability, fear, anxiety, anergia, pain, and insomnia (Kraepelin, 1907; Bleuler, 1949; Mula et al., 2008).

In 1986, Mendez et al. (1986), in a sample of outpatient patients with epilepsy and of matched controls, investigated the prevalence of depression using DSM-III-R criteria. They found that in the majority of patients with epilepsy they were affected from depression, reported prior suicide attempts and were four times more likely to have been hospitalized for depression compared to non-epileptic patients. Patients with epilepsy and depression presented the characteristics of the “endogenous” features of depression, characterized by psychotic traits and dysthymia. Therefore, a specific subtype of epilepsy–due to an alteration in the limbic system–was described. Blumer named such clinical condition as “interictal dysphoric disorder”, Such condition has chronic course, with recurrent symptom-free periods and can be treated with low doses of antidepressant medication. Kanner described another specific subtype of clinical depression reported by patients with epilepsy, called “dysthymic-like disorder of epilepsy”. In these patients, the clinical condition is characterized by anhedonia, hopelessness, fatigue, anxiety, irritability, lack of concentration, frustration, mood lability and alteration in appetite and sleep patterns.

Kanner described also a “subsyndromic” subtype of depression in patients with epilepsy (Kanner, 2006), characterized by patients failing to fulfil the DSM-IV criteria for major depressive disorder, but reporting symptoms of depression of mild-to-moderate severity, anxiety, irritability, physical symptoms and increased energy, with a significantly reduced levels of quality of life.

#### Classification based on the onset of depression compared to the seizure

Based on the onset of depression compared to the seizure, different types of depression have been identified including: 1) Preictal; 2) Ictal Focal; 3) Postictal; 4) Interictal. Among these subtypes, interictal depression is the most common (Mula et al., 2008; Kanner et al., 2024; Kanner and Palac, 2000).

People with epilepsy can develop depressive symptoms around the epileptic seizure, which are defined as peri-ictal symptoms or as a side effect of the antiseizure treatment (i.e., drugs or surgery). For the appropriate management and treatment of depression in patients with epilepsy, it is essential to identify the different factors contributing to the disorder. In fact, each of these domains can be treated using different and integrated approaches [including psychotherapy, counselling, antidepressant medications, antiseizure medication (Mula, 2019)]. Depression in epilepsy can occur in a number of different clinical contexts and not just as a comorbid disorder. Depression is reported more frequently in patients with temporal lobe epilepsy and left-sided foci, although prevalence data are controversial (Lothe et al., 2008).

### Screening for depression in patients with epilepsy

Diagnosing depressive disorders in people with epilepsy can be challenging due to the complex inter-relationship between these two clinical conditions.

Therefore, it is essential in ordinary clinical practice to consider the following risk factors: seizure severity and frequency, recent changes in ASMs, number and symptoms of depressive episodes, and family history of psychiatric illness (particularly depression), which are associated to the heightened risk of developing a depressive episode.

Epilepsy with comorbid depression may present with sub-syndromic depressive episodes and clinicians should be able to perform a differential diagnosis for other psychiatric conditions, including anxiety disorders, psychotic disorders, bipolar disorders as well as should be able to detect any side-effect due to drug interactions or as a consequence of epilepsy surgery.

Therefore, it is essential to use in ordinary clinical practice validated screening tools, in order to promote an adequate and prompt detection of depressive, anxiety, and suicidal behaviours in people with epilepsy (Fiorillo et al., 2018). Screening tools only provide data that must then be carefully considered by trained personnel. Patients positive at the screening must undergo a complete mental state examination by a psychiatrists, in order to formulate an appropriate diagnosis.

Several assessment tools have been developed in order to promptly detect the presence of depressive symptoms, including the 6-item Neurological Disorders Depression Inventory for Epilepsy (NDDI-E); the Emotional Thermometer (ET); and the 21-item Liverpool Adverse Events Profile (LAEP) (Table 1). The routinary adoption of this tool has been recommended by a consensus of the Mood Disorder Initiative-Epilepsy Foundation. In particular, a score of 15 or above at NDDI-E is suggestive of a major depressive episode, but a recent meta-analysis by Kim et al. (2018) found that the optimal cutoff for detecting major depression is >13. The NDDI-E and ET have a high negative predictive value with a low positive predictive value. The low scores indicate a negative assessment and are highly reliable. A score higher than the cut-off scores may potentially indicate a positive assessment of depression but it requires an in-depth clinical assessment for confirmation. The most frequently used and developed tool targeting depression in people with epilepsy is the six-item Neurological Disorders Depression Inventory for Epilepsy (NDDI-E).

**TABLE 1:** Main assessment tools for detecting the presence of depressive symptoms and/or suicidal ideations in patients with depression and epilepsy.

Name of the scale	Acronym	Description
Neurological Disorders Depression Inventory for Epilepsy	NDDI-E	Self-administered instrument consisting of 6 items rated by the patients on a balanced four-point scale ranging from “never” (score = 1), “rarely” (score = 2), “sometimes” (score = 3), to “always or often” (score = 4) and takes less than 3 min to complete The score is obtained by computing the sum of the scores obtained by items. The possible overall score ranges from 6 to 24
Emotional Thermometer	ET	A self-report, pencil and paper measure consisting of a line with a 0 to 10 scale anchored at the zero point with “No Distress” and at scale point ten with “Extreme Distress”
Liverpool Adverse Events Profile	LAEP	The K-LAEP is a 19-item self-report questionnaire used to identify and monitor the frequency and severity of common adverse effects associated with AEDs in Korean PWE. This questionnaire evaluates the frequency of adverse effects of antiseizure medications occurred during the past 4 weeks. Each item is scored on a four-point Likert scale: 1, never a problem; 2, rarely a problem; 3, sometimes a problem; and 4, often or always a problem. The total score ranges from 19 to 76, and higher scores are indicative of a greater burden from the adverse effects. The Cronbach’s α coefficient of the K-LAEP is 0.9
Intericatal Depression Inventory	IDI	
Patient Health Questionnaire-9	PHQ-9	A self-administered tool, consisting of 9 items, each rated on a Likert scale from “0” (not at all) to “3” (nearly every day)
Suicidal Ideation Attribution Scale	SIDAS	It is a self-reported tool, aiming to screen individuals for presence of suicidal thoughts and assess the severity of these thoughts. It consists of five items, each targeting an attribute of suicidal thoughts: frequency, controllability, closeness to attempt, level of distress associated with the thoughts and impact on daily functioning. Responses are measured on a 10-point scale. Items are coded so that a higher total score reflects more severe suicidal thoughts
Columbia Suicide Severity Rating Scale	C-SSRS	The scale is intended to be used by individuals who have received training in its administration. The administrators ask a series of questions about suicidal thoughts and behaviors. The number and choice of questions they ask depend on each person’s answers. The questioner marks “yes” or “no,” as well as how recently the thought or behavior occurred and a scoring of its severity
Hospital Anxiety and Depression Scale	HADS	The HADS is a self-report rating scale of 14 items on a 4-point Likert scale (range 0–3). It is designed to measure anxiety and depression (7 items for each subscale). The total score is the sum of the 14 items, and for each subscale the score is the sum of the respective seven items (ranging from 0 to 21)
Beck Depression Inventory-II	BDI-II	A 21-item self-report inventory measuring the severity of depression in adolescents and adults
Toronto Alexithymia Scale	TAS-20	It is a 20-item self-report questionnaire that can be used to identify issues relating to alexithymia such as difficulty recognising, describing and regulating internal emotional states

Moreover, clinicians should consider administering to the patients the following screening tools such as the Patient Health Questionnaire-9 (PHQ-9), the Hospital Anxiety and Depression Scale (HADS), the Beck Depression Inventory-II (BDI-II) and the Toronto Alexithymia Scale (TAS-20).

Clinicians should carefully evaluate suicidal ideation and suicidal risk, also considering that the risk of suicide in patients with epilepsy is high, regardless of the presence of depression. Therefore, screening for suicidality should be an integral part of the evaluation of every patients with epilepsy during all follow-up period. Several assessment tools are available such as the Suicidal Ideation Attribution Scale (SIDAS) or the Columbia Suicide Severity Rating Scale (C-SSRS), which require an *ad hoc* training in order to be administered should be considered. However, it is essential to carefully evaluate the personal and family history in order to detect potential risk factors for suicide/suicidal ideation (Table 1).

### Management plan for persons with comorbid depression and epilepsy

The management plan of patients with comorbid depression and epilepsy is complex and requires a strong collaboration among different specialties. In some cases, patients can present a personal history positive for other mental disorders, further highlighting the need for a strong collaboration between neurologists and psychiatrists.

In particular, the management plan of patients with epilepsy and depression should include pharmacological and non-pharmacological interventions (Schumacher et al., 2024; Luciano et al., 2012), based on the clinical characteristics of each individual patient (Leichsenring et al., 2023; Arroll et al., 2023; Hasler, 2023; Sampogna et al., 2024). As regards the selection of ASM, it will depend on the characteristics of the disease and the patient’s profile. A recent consensus document by Villanueva et al. (2023) has suggested to prefer the use of lamotrigine, valproate, carbamazepine, oxcarbazepine or eslicarbazepine acetate. The drug selection should be based on seizure type, age, gender, presence of comorbidities, other medications and childbearing potential (Yang et al., 2024; Brodi et al., 2016; Maguire et al., 2021).

**FIGURE 2:**
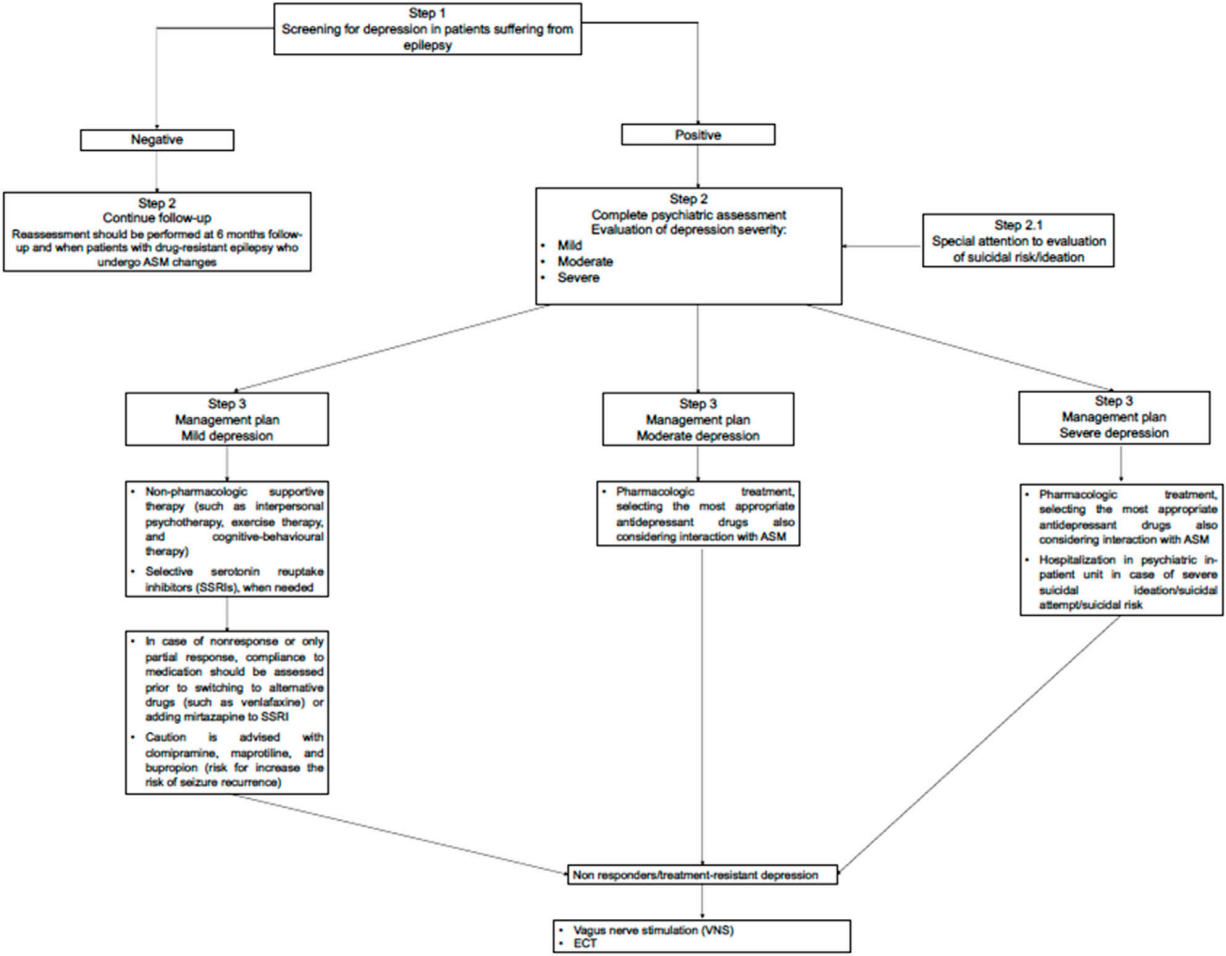
Flowchart for the management of depressive symptoms/comorbid depression in people with epilepsy.

As regards the treatment of depression in patients with epilepsy, clinicians should carefully assess the severity of the depressive symptoms, formulate the diagnosis and assess the presence of suicidal ideation and of suicidal risk (Figure 2).

Therefore, for all patients suffering from epilepsy and comorbid depression, it should be advisable to start treatment for depression as soon as possible. A dedicated attention should be paid to patients at high suicidal risk, even considering hospitalization in psychiatric units when the suicide risk is rated as very high. Reassessment for depression and suicidality should be performed at baseline and at 6 months follow-up in patients with drug-resistant epilepsy who undergo ASM changes. In particular, clinicians should consider that up to 6% of patients scored negative at baseline for suicidal ideation, when re-assessed after 6 months report significant levels of suicidal ideation. Furthermore, this risk is increased in people already positive at baseline for the presence of psychiatric comorbidity (Mula et al., 2024).

The treatment for mild depressive episode should include non-pharmacologic supportive therapy (e.g., interpersonal psychotherapy and cognitive-behavioral therapy) as a first-line treatment (Mula et al., 2022) associated with selective serotonin reuptake inhibitors (SSRIs) when needed. The pharmacological regimen including antidepressants should be maintained for at least 12 months, depending on the presence of previous depressive episodes, the type of response to antidepressant treatment. The discontinuation should be considered taking into account the time from when the patient remits at least 50% of acute depressive symptoms, since, SSRIs have a 4–6-week latency period, which can extend the total treatment time. Furthermore, it is necessary to consider whether the patient is suffering from a first episode or if it is a relapse/recurrence). Therefore, pharmacological discontinuation should be considered according to a stepwise model (Mula et al., 2022).

In case of nonresponse or only partial response, compliance to medication should be assessed prior to switching to other type of antidepressants (such as SNRI, e.g., venlafaxine) (Mula et al., 2022) or adding mirtazapine to SSRI. Caution is advised with clomipramine, maprotiline, and bupropion, as these drugs may increase the risk of seizure recurrence.

Clinicians should carefully consider and manage the possible pharmacodynamic and pharmacokinetic interactions among antidepressant drugs and ASM (as reported in Tables 2, 3). In particular, clinicians in selecting the most appropriate antidepressant drug should considered that when using SSRI, fluoxetine and paroxetine act as cytochrome 2D6 inhibitor and therefore can potentiate the toxicity related to ASM. It should be considered that among ASM also carbamazepine acts cytochrome 2D6 inhibitor.

**TABLE 2:** Drugs interacting at cythocrome P450 2D6.

		Inhibitors		Inductors	
Antidepressants	Amitriptiline	Antidepressants	Amitriptiline	ASM	Carbamazepine
	Clormipramiea		Clorimipramine		
	Citalopram				
	Doxepina		Fluvoxamine		
	Fluoxetine		Flufenazine		
	Imipramine		Nefazodone		
	Nortriptiline		Paroxetine		
	Paroxetine		Sertraline		
	Venlafaxine		Venlafaxine		
	Mirtazapine	Antipsichoticis	Haloperidol		
Antipsichotics	Olanzapine		Perfenazine		
	Mirtazapine		Tioridazine		
	Clozapine				
	Haloperidol				
	Perfenazine				
	Risperidone				
	Tioridazine				

**TABLE 3:** Drugs interacting at cythocrome P450 2C9.

		Inhibitors		Inductors	
ASM	Fenitoine	Antidepressants	Fluoxetine	ASM	Carbamazepine
	Valproic acid		Fluvoxamine		Fenitoine
Antipsychotics	Clozapine				
Antidepressants	Amitriptiline				
	Fluoxetine				
	Sertraline				

Caution should be applied when ASMs, are changed in female patients in preparation for pregnancy. For example, changing carbamazepine or another ASM, that has teratogenic potency to levetiracetam includes the risk of developing subsequent depression (Gammoh et al., 2024).

Clinicians must be vigilant and promptly detect mood swings or the presence of suicidal ideation, no delay treatment with ASMs or with antidepressant drugs is recommended.

The treatment of epilepsy represents a challenge due to the complexity and diversity of the mechanisms associated. Although diagnostic techniques and pharmacological treatment have been improved, there are still more patients that do not obtain a clinical remission. In particular, up to one-third of patients with epilepsy unsuccessfully respond to drug treatment, suffering from drug-resistant epilepsy (Medel-Matus et al., 2022). Patients with drug-resistant epilepsy can obtain a seizure remission period lasting for years, but more than 70% of them experience a relapse in the 12-month post-remission period. In such subgroup of patients, it is extremely relevant to detect the presence of psychiatric comorbidities, since those comorbid conditions can reduce their impact on the development of this condition and improve their response to pharmacological treatment. Moreover, in patients with drug-resistant epilepsy it is particular relevant to detect depressive symptoms, since these are significant predictors of poor quality of life and of suicidal ideation.

Vagus nerve stimulation (VNS) may be an effective palliative therapy in drug-resistant epileptic patients and it is also approved as a therapy for treatment-resistant depression. VNS may ameliorate depressive symptoms and its effect is uncorrelated to seizure response (Assenza et al., 2020). VNS may be considered for treatment-resistant depression, keeping in mind that the optimal parameters for VNS in depression may differ from those used for epilepsy (Nickels, 2021).

The use of electroconvulsive therapy (ECT) in patients with epilepsy is still controversial (Asadi-Pooya, 2017). ECT has been employed as a treatment for refractory epilepsy and status epilepticus in a few anecdotal reports, sometimes successfully. A recent scoping review highlighted that the current existing evidence have some limitations. Clinicians should carefully assess the individual case and evaluate the risk/benefit ratio for using ECT (Ong et al., 2024). If patients present suicidal thoughts or intent, they should be referred to a psychiatrist for an in-depth assessment. In most severe case, it should be considered a psychiatric hospitalization in order to protect patient’s life (Nickels, 2021). When patients with epilepsy and comorbid depression develop psychotic features, a referral to mental health centre is necessary, in order to develop a personalized treatment plan including both antidepressant and antipsychotic medication.

Another complex condition to be managed is represented by Psychogenic Non-Epileptic Seizures (PNES), defined as paroxysmal changes in behavior, consciousness and autonomic function that resembles epileptic seizures, without any electroencephalographic (EEG) alterations of epileptic seizures (Avalos et al., 2020; Berking, 2024). These clinical conditions have been recently redefined as “functional seizures”. This definition has been abandoned since it has always been perceived as stigmatizing by patients, carers and professionals themselves (Vilyte and Pretorius, 2019; Stone et al., 2024; Creed, 2023). Patients suffering from functional seizures can take an average of 7 years between the manifestation of clinical symptoms and definite diagnosis. In some cases, patients with functional seizures present a comorbid depressive disorder, which further complicate its clinical management (Jafari et al., 2020).

There is a limited knowledge regarding the best management strategies for functional seizures and comorbid depression. Several non-pharmacological and pharmacological interventions have been proposed, based on the stage of the disorder. In particular, in the initial phase of treatment (diagnosis delivery phase), effective collaboration and communication between psychiatrists and neurologists is very important, since patients should have received many diagnoses over time. An Italian multidisciplinary Consensus-Based Standard (CBS) systematic review (Gasparini et al., 2019) suggest a multidisciplinary approach to patients with functional seizures. The board recommended to carefully assess individuals with functional seizures for mood disorders, personality disorders, and psychological trauma. Cognitive behavioral therapy should be considered the first-line psychological treatment, with pharmacological interventions used to manage co-occurring conditions such as anxiety and depression.

## Discussion

The complexity of comorbidity of epilepsy and depression represents a challenge for neurologists and psychiatrists, in terms of correct detection and management of these clinical conditions. Both epilepsy and depression represent severe chronic disorders, associated with a significant level of personal and social impairment, with often an underestimated prevalence of these conditions, due to stigma and misconceptions attached to these diseases. Therefore, the management of comorbid epilepsy and depression requires a detailed clinical characterization of each individual case in order to develop an integrated and personalized management plan.

Several unmet clinical needs in the management plan of patients with comorbid depression and epilepsy have been highlighted according to psychiatrists and neurologists’ viewpoint.

In particular, according to psychiatrist’s viewpoint patients with comorbid epilepsy and depression represent a challenge for daily clinical care due to the complexity in detection, diagnosis, treatment and long-term management plan (Graham-Rowe et al., 2023; Fiorillo et al., 2013). The main unmet needs reported by psychiatrists include the need to establish and strengthen a network of collaboration with neurologists in order to collaborate actively in the whole process.

In particular, the establishment of a collaborative network should accelerate the pathway to diagnosis and reduce the help-seeking delay, which should be associated with a significant improvement in patients’ quality of life. Moreover, psychiatrists highlight the fact that patients with epilepsy tend to overlook any mood or psychiatric symptoms in general, attributing all these phenomena to the underlying epileptic condition. A significant factor contributing to this should be represented by the lack of mental health literacy in the general population and by the presence of stigmatizing attitudes and behaviours towards mental health issues (Henderson, 2023; Hart et al., 2023). There is the need to promote a cultural change in the general population, as well as in the healthcare section, in order to promptly detect any mental health problem as soon as possible.

Another clinical unmet need is represented by the few data available on treatment of depression in epilepsy. Consensus documents have been released with some recommendation, but the level of evidence is still not satisfying, compared to those available for the management and treatment of patients suffering from depression or from epilepsy, as single disease (Mula, 2019; Kerr et al., 2011; Barry et al., 2008).

Taking a multidisciplinary approach to treating a mood disorder in a patient who has epilepsy might improve epilepsy and depressive disorder’s outcomes. Moreover, it is necessary to promote a shared decision-making process between clinicians and patients in order to improve patients’ outcomes (Clarke et al., 2015; Bär Deucher et al., 2016; Puschner et al., 2010).

According to neurologist’s viewpoint, the timing of the symptoms and occurrence of recent seizure activity can be used to classify depressive symptoms as preictal, ictal, postictal, or interictal. The most important clinical unmet need for neurologists is represented by the promptness of diagnosis of depressive symptoms and/or depressive episodes. Often, neurologists prioritize collecting the epileptological history leading to limited time available for a thorough investigation of potential co-occurring depressive symptoms.” Although there are assessment tools available–such as the Neurologic Disorder Depression Inventory-Epilepsy (NDDIE), which is validated tool for assessing the presence of depression and suicidality in people with epilepsy, the sensitive of the tool is somewhat limited. In particular, the NDDIE is a 6-question rating scale that is self-compiled by the patient, but it can be also scored by the clinician or be administered and scored during the appointment. However, it should be advisable to refer the patient to a specific consultation with a psychiatrist, in order to formulate a full-blown diagnosis. Although the best practice after a positive screening is to collaborate with a psychiatrist for further assessment and treatment, in some cases, this should not be possible due to a fragmentation of healthcare system.

Moreover, another relevant issue is represented by the misconception and the low level of health literacy found in the general population on epilepsy and depression. The level of stigmatization attached to epilepsy and depression is particularly high in specific culture, such as Sub-Saharan Africa, due to superstitious cultural and traditional beliefs for explaining the causes of epilepsy (Singh et al., 2018). These misconceptions can delay the access to care for epilepsy and–in turn–can have a detrimental impact on the detection of depressive symptoms in people with epilepsy.

Collaborative care has been proposed as the best way to deal with comorbidity and where it is possible to establish such a system people with comorbid conditions usually receive care which they need. Unfortunately, in many places collaborative care has not established yet and even in places in which it has been put in practice, it is made difficult because of the separation between institutions providing care for people with mental disorders and for those suffering from other disorders (Sartorious, 2013; Fiorillo and Sartorius, 2021; Momen et al., 2024). Moreover, the “fragmentation” of care and of specialties, which affects almost all professions–including medicine, represents a further obstacle for the appropriate management of patients suffering from comorbid, complex conditions such as depression and epilepsy. Therefore, an integrated model of care should be advocated by healthcare professionals in order to provide the best care to patients with epilepsy and comorbid depression, in order to develop a management plan tailored to their needs, psychiatric history, disease characteristics (Fiorillo and Giordano, 2022; Sartorious, 2013; Zachar, 2023).

The reality of clinical work in outpatient and inpatient psychiatric and neurological units is very different, with a limited access to psychiatric care, and many patients with epilepsy and comorbid depression are treated by neurologist.

However, it should be necessary to promote a correct information at the level of the general population, particularly for patients and their caregivers, about the prevalence of depressive symptoms in people with epilepsy as well as to improve educational activities for healthcare professionals, in order to disseminate screening procedure for depressive disorders and to further strengthen an integrated care management between psychiatrists and neurologists. Complex and challenging clinical conditions, such as epilepsy with comorbid depressive disorder, necessitate a biopsychosocial perspective and a collaborative, multidisciplinary strategy to ensure optimal patient management and care (Özge et al., 2023).

Moreover, depressive symptoms in people with epilepsy are often present by the time of the first recognized epileptic seizure, further highlighting the need for psychiatric screening as soon as possible, especially at the initial stages of the care process of patients with epilepsy. An in-depth psychiatric assessment should be considered also because patients may present other psychiatric comorbidities, such as anxiety disorders, attention deficit and psychotic disorders and less frequently, personality disorders.

The need for a closer collaboration between psychiatrists and neurologists Is confirmed also by the management plan to be issued for each patient. In particular, the selection of a specific antiseizure medication should consider not only its safety and tolerability, but also the risk of increasing suicidal risk or improving quality of life. Therefore, it is essential to specifically assess these dimensions before starting the management plan, and only a collaboration between psychiatrists and neurologists can work toward such global aim.
